# Fungal Extracellular Vesicle Proteins with Potential in Biological Interaction

**DOI:** 10.3390/molecules29174012

**Published:** 2024-08-24

**Authors:** Jingyan Xu, Yujin Zhao, Yanguang Zhou, Shijie Dai, Na Zhu, Qingling Meng, Sen Fan, Weichun Zhao, Xiaofeng Yuan

**Affiliations:** College of Life Science, Zhejiang Chinese Medical University, 548 Binwen Road, Binjiang District, Hangzhou 310053, China; 15824357597@163.com (J.X.); zhaoyujin326@163.com (Y.Z.); zhouyanguang07@163.com (Y.Z.); dsj2513140@163.com (S.D.); zhuna85@163.com (N.Z.); 15192866713@163.com (Q.M.); fansen2001@163.com (S.F.); weichunzhao@zcmu.edu.cn (W.Z.)

**Keywords:** fungi, extracellular vesicles, protein, transmembrane, communication, role

## Abstract

Extracellular vesicles (EVs) are vesicle-like structures composed of lipid bilayers, which can be divided into apoptotic bodies, microbubbles and exosomes. They are nanoparticles used for the exchange of information between cells. EVs contains many substances, including protein. With the development of proteomics, we know more about the types and functions of protein in vesicles. The potential functions of proteins in the envelope are mainly discussed, including cell wall construction, fungal virulence transmission, signal transmission and redox reactions, which provides a new perspective for studying the interaction mechanism between fungi and other organisms. The fungal protein markers of EVs are also summarized, which provided an exploration tool for studying the mechanism of vesicles. In addition, the possible role of immune protein in the EVs in the treatment of human diseases is also discussed, which provides new ideas for vaccine development.

## 1. Introduction

**Figure 1:**
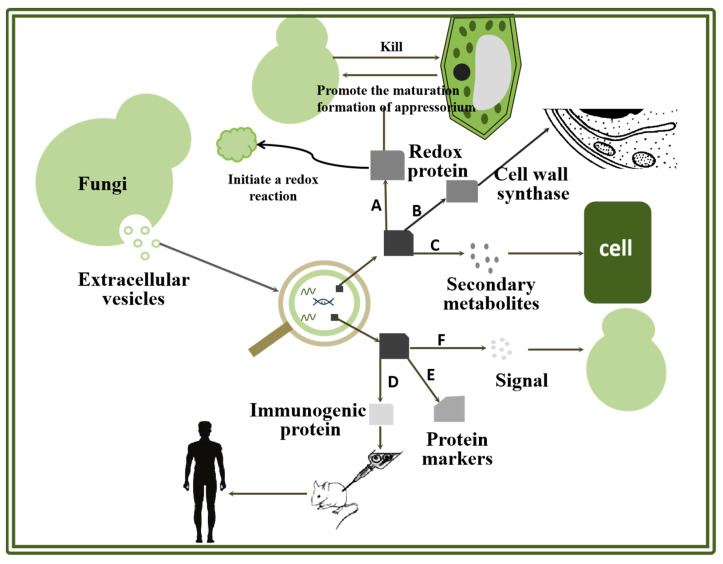
Protein function in fungal EVs. A. Role of protein in oxidation reaction. B. Role of protein in cell wall synthesis. C. Role of protein in virulence transmission. D. Role of protein in vaccine research and development. E. Role of protein in the study of markers. F. Role of protein in transmission.

**Figure 2:**
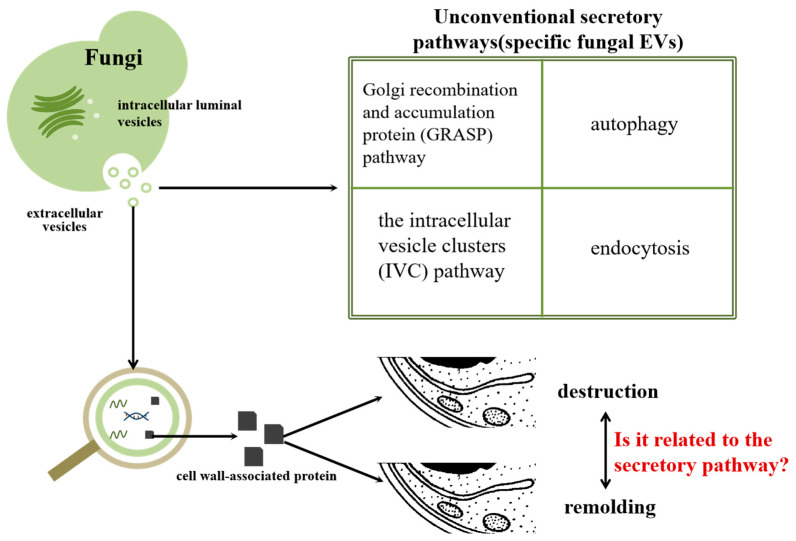
Possible secretory mechanism of fungal EVs and function speculation of cell wall-related proteins in vesicle.

EVs play an important role in cell transmembrane communication, and there are significant differences among different fungi [1,2,3,4,5]. The vesicles may be released through the Golgi reorganization and accumulation protein (GRASP) pathway, autophagy, the intracellular vesicle cluster (IVC) pathway and plasma membrane budding [5,6,7] (Figure 1 and Figure 2). Interestingly, the content of protein in the EVs of protoplasts and fungi with intact cell walls will be different, and the content of EVs extracted from protoplasts and the protein type have changed. The secretion of vesicles may be related to the cell wall. In the process of vesicle secretion, the exchange of protein, lipids and other substances can be realized by EV as a medium, so as to achieve the purpose of transmembrane regulation. There is evidence that EVs can be absorbed by fungal cells. Containing GPI anchor protein and AAA domain, it can be used to modify the protein transported in vesicles and participate in membrane fusion. This indicates that EVs can transport protein into other biological cells and participate in interactions between organisms [8,9,10]. In many organisms, protein in vesicles has been proven to have many biological functions [11,12,13]. Fungal EVs can inhibit the formation of fungal hyphae and biofilm, thus weakening the virulence of fungi and inhibiting their growth [14]. However, some researchers have found that fungal EVs can promote the growth of hyphae [15], so it is believed that the EVs of different fungi have different effects on the growth of mycelia. At the same time, it was found that EVs shared between yeast cells contributed to heat tolerance [16]. In the process of interaction between fungi and other organisms, the EVs of human pathogenic bacteria such as *Candida albicans* can promote infection and inflammatory reaction, play a role in the pathogenic mechanism of fungi and cause host immune response [17,18]. EVs not only play a role in pathogenesis, but also promote the anti-oxidation and anti-radiation ability of cells, thus improving the prevention of oxidative stress damage induced by infrared rays and reducing the occurrence of diseases [19]. In other organisms, such as parasites, EVs have the effect of co-evolution with other organisms [20]. EVs play different roles in different organisms, which shows their value in mechanism research.

After EVs are produced by fungi, their functions are mediated by specific loading proteins [21]. The protein in the EVs is transferred to the subject by fusion with the plasma membrane, which requires the participation of SNARE proteins [22,23]. With the development of proteomics, the EVs of many fungi have been thus identified. Although there is no in-depth study on the specific mechanism at present, according to the current data, endoproteins have different functions, including cell wall construction, virulence transmission and signal transmission (Figure 1). Based on these data, protein plays an important role in the formation and function of EVs, which can provide a new angle for studying the mechanisms of fungi and help to make better use of fungi to reduce and treat diseases.

Fungal function is closely related to the mechanism of action.Proteomics show that most of the EVs produced by fungi contain protein [24,25,26]. Cell wall synthesis-related protein, redox-related protein, protein related to secondary metabolites and virulence-related protein have been found in many fungal EVs, which indicates that EVs play an important role in cell wall synthesis and affect the redox reaction and toxicity to fungi [27,28]. At present, the research methods of mammalian EVs have matured and been established, there are very effective EV-specific markers [29,30], and EVs have played a role in disease surveillance because they overcome natural barriers and targeting [31,32]. In the preliminary study of fungal EV markers, research methods regarding mammalian EV markers were used for reference, which delayed the study of fungal EV markers [33,34,35]. Until recent decades, the study of protein markers in yeast and filamentous fungi achieved Initial results, and the study of fungal EV markers has gradually entered the right track [33,36]. It is worth noting that the proteins in the EVs of some fungi are immunogenic, and it has been found that these EVs can cause an immune response in mice, which is similar to that of vaccines [37]. The application of EVs provide a new way for the treatment of fungal diseases.

## 2. The Different Proteins of EVs in Fungi

### 2.1. Role of Proteins in Cell Wall Synthesis

The components of fungal cell walls are different, but most of them show similar structures. The cell wall is mainly composed of polysaccharides and highly glycosylated protein. Cell wall-related proteins are very important in the process of cell wall formation. Researchers have found cell wall-associated proteins in EVs (Figure 1B). They speculate that protein in EVs participates in biological processes, such as the increase of chitin in the cell wall and the change of osmotic pressure and cell wall pressure, and plays an important role in the construction of fungal cell walls [38,39,40,41].

Through proteomics and other analytical methods, it was found that more than ten kinds of fungal EVs reported at present all contain protein related to cell wall synthesis and remodeling. Enzymes necessary for the synthesis and degradation of chitin, α-1, 3-glucan and β-1, 3-grape glycation, have been detected in the EVs of many fungi, including *Botrytis cinerea*, *Trichoderma harzianum*, *Colletotrichum higginsianum*, *Aspergillus fumigatus* and *Fusarium* [36,42,43,44,45]. The secretion of enterovirus is closely related to the cell wall. In the process of secretion, the EVs may interact with the cell wall. The protein in the vesicle also plays a certain role in the cell wall, but the specific mechanism has not been reported.

Protein related to cell wall synthesis has also been found also found in the study of four pathogenic fungi that threaten human life and health, namely *Candida albicans*, *Cryptococcus capsulatus* and *Paracoccospora brazil*. EVs have also been proven to improve the drug resistance of cell wall deletion mutants, so it is believed that cell wall proteins also interact with cell walls when they secrete EVs [25,37,46,47,48]. Proteomic analysis of the non-pathogenic fungus Saccharomyces cerevisiae showed that there were proteins involved in cell wall remodeling in vesicles [49]. Although many other fungi that threaten the health of plants, animals and humans have not been reported on, it is inferred from the existing data that their vesicles should contain cell wall-related protein. Based on the data regarding proteins in EVs of various fungi, we think that there is a strong correlation between the secretion of EVs and cell walls. Vesicles are secreted through cell walls by the fusion–separation method. During this period, EVs containing cell wall-related proteins can be secreted out of fungal cells by destruction and reconstruction, and their functions can be exerted (Figure 2).

### 2.2. Role of Protein in Virulence Transmission

Protein related to metabolic pathways in fungi will affect the occurrence and development of glycolysis, gluconeogenesis and the tricarboxylic acid cycle and play an important regulatory role in fungal function and some infection processes [50] (Figure 1C), and these metabolism-related proteins were also detected in EVs [51,52]. Moreover, fungi can affect other species by secreting toxic secondary metabolites [53,54]; for example, *Aspergillus flavus*, *Aspergillus clavatus*, *Aspergillus hirsutus* and other fungi of the genus Aspergillus all produce corresponding mycotoxins, which pose a great threat to humans and animals [55]. Secondary metabolites secreted by endophytic fungi, when interacting with plants, help fungi avoid plant defense and achieve colonization in plants [56] to achieve the purpose of causing disease. The familiar effectors, toxins, cell wall degrading enzymes, organic acids, etc. can all be used as virulence factors of fungi. In fact, proteins can also be used as virulence factors to participate in the process of pathogen infection [57]. When EVs interact with other organisms, virulence-related proteins are transferred to the cells, so that the virulence of fungi can be transmitted across the membrane, thus achieving the purpose of causing diseases.

As an important medium of fungal interaction, EVs play a vital role in the development of fungal virulence. In the interaction between fungi and plants, the EVs of *Fusarium oxysporum* can cause cotton fungal infection. From the point of view of protein molecules, it has been found that the protein related to the disease was significantly enriched. For example, many virulence-related proteins, such as polypeptide synthetase, MAP kinase, and FMK and GMP synthetase, are all necessary for toxin production. Therefore, it is speculated that vesicles can be used as a medium to transport protein in plants for the purpose of infection [58]. Proteomics shows that there are proteins related to the production of polyketone, non-ribosomal peptide, alkaloids and terpene secondary metabolites in the EVs of *Colletotrichum gossypii*, and these secondary metabolites are usually related to virulence, which can help fungi to cause disease. This also proves to some extent that EVs also have a pathogenic function, and this function cannot be carried out without its internal protein [44]. GAG has been detected in the EVs of Aspergillus fumigatus, which is a virulence-related component of the extracellular matrix. However, there are still few descriptions of the virulence-related proteins in Aspergillus fumigatus, and their related functions have not been reported in detail [45].

*Candida albicans* is a common human pathogen. Researchers have carried out in-depth research on the pathogenesis of its cells, but more data are needed to support the study of its vesicle function. At present, enzymes related to the ability to activate plasminogen have been found in the vesicles of *Candida albicans*, while in fungi, plasminogen binding protein is a non-specific opportunistic pathogenic factor, and this kind of protease can make fungi more likely to achieve the purpose of pathogenicity [59,60]. In addition, Sap family proteins have also been detected in the vesicles of *Candida albicans* ATCC 900. It is known that the functions of these family proteins in cells are closely related to pathogenesis, and the detection of these proteins in vesicles can also confirms the important role of vesicles in pathogenesis. The EVs of *Cryptococcus* are considered the key to the formation of virulence mechanisms, and urease, a virulence substance detected in fungal cells, also has high activity in EVs [61,62]. At present, there are no pathogenic proteins in yeast, so only some studies have preliminarily explored the pathogenicity of fungal EVs, but a lot of data are still needed to prove their function. The role of these proteins may be that virulence factors directly act on the host cells through the EVs and that toxins are synthesized by metabolism-related proteins to act on the host cells. Researchers are gradually exploring virulence-related proteins in EVs. Through a protein omics study, we found that there are many virulence proteins in EVs, but the specific mechanism of their action needs to be further explored. However, it is undeniable that the proteins in EVs definitely play a role in the pathogenic processes of fungi.

### 2.3. Role of Protein in Transmitting Information

Fungi release EVs to realize information transmission [63]. Protein has the function of signal transmission in biological interaction. It can transmit signals to fungi according to different environmental factors and adjust the physiological state of fungi [64]. EVs include protein, RNA and other signaling molecules. EVs are the key determinant of intercellular communication, which has the functions of material exchange and signal transmission in the process of information transmission [65,66].

The protein-recognizable signal in EVs is an important medium in the process of inter-species communication (Figure 1F). The membrane protein on EVs internalizes the contents of EVs by binding with the receptor protein, so as to realize information transmission between cells [67,68,69,70]. In drug delivery platforms, EVs also play an increasingly important role in the treatment of diseases. EVs from grapefruit, lemon and other plants can play a transfer role in targeted cancer therapy through internalization [71], which provides a new idea for precise targeted treatment of diseases.

In addition to plant cells, protein, which can transmit information, has also been found in the EVs of fungi. The EVs of symbiotic yeast *Malassezia sympodialis* play a role when they come into contact with the skin, and they contain a rich variety of protein [70]. HSP70 protein was detected in *Aspergillus fumigatus, Fusarium oxysporum* and *Cryptococcus* EVs infected with plants [72,73,74]. When plants are infected by fungi, this protein transmits immune information to the pathogen, making it produce a series of defensive reactions to protect itself. In the process of infection, fungi will release EVs, which play a role in information transmission in the process of fungal invasion and defense, thus realizing the interaction between organisms.

### 2.4. Role of Protein in Oxidation Reaction

The oxidation reaction is related to reactive oxygen species (ROS) and reactive nitrogen species (RON) and plays a role in the interaction between different species [74,75,76] (Figure 1A). The antioxidant system plays an important role in detoxifying and maintaining plant health by balancing redox reactions. In plants, the content of ROS needs to be maintained at a normal level. Excessive ROS will lead to lipid peroxidation and even cell death. Studies have confirmed that abiotic stresses such as nano-silver, salinity and so on will affect the oxidation of plants [77,78]. Oxidation also plays an important role in the occurrence of cancer, and during this period, the EVs of cancer cells are always active [79] by changing the tumor microenvironment (TME) to promote tumor progress, thus promoting tumor growth and survival [80].

It is worth noting that reactive oxygen species (ROS) play a key role in the interaction between plants and pathogens. For fungi, reactive oxygen species can promote the formation and maturation of adherent cells; for plants, reactive oxygen species can directly kill fungi, strengthen cell walls or induce signal pathways [81,82]. Mitochondria are the main source of ROS, and EVs are rich in mitochondria and mitochondrial components, which can be transported through vesicles to reach target cells. At present, there are two main ways for EVs to affect redox. First, mitochondria act directly on target cells, affecting their mitochondrial function; second, the function of mitochondria in target cells is regulated by the components in vesicles [83]. As a carrier of protein, protein related to oxidation and reduction has been detected in the fungal EVs, so it was believed to be related to oxidation. Therefore, EVs bear the antioxidant capacity of fungi and play an important role in fungal defense as a scavenger of reactive oxygen species [84].

In fungi, it has been found that *Candida albicans* EVs can promote the oxidation reaction, and they are rich in oxidase and ROS molecules, which affects the oxidative damage. It is speculated that the oxidation-related proteins in fungal EVs are closely related to the redox reaction function of fungi [85]. Another study found that EVs can inhibit the expression of redox-related genes and reduce the level of ROS in cells, which leads to cell aging and death and plays a role in fungal pathogenesis [86]. Interestingly, the EVs of Morchella also have the function of inhibiting ROS production and then inhibiting the severe stress response. Due to the lack of protein omics data, it is impossible to analyze the protein in *Morchella* vesicles [87]. In a few reported fungi (*Histoplasma capsulatum*, *Paraspora*, *Fusarium oxysporum*, *Fusarium graminearum*, etc.) redox-related proteins have been detected by proteomics [25,34,36]. However, due to the lack of in-depth study of its mechanism, we can only confirm that it exists in vesicles, and further research is needed to explore its function. The oxidation reaction of fungi is an important reaction for fungi, and there are related proteins in vesicles, which may provide a new perspective for studying the interaction mechanism between fungi and other organisms, enrich the functional research of the EVs and make better use of EVs.

### 2.5. Role of Protein in the Study of Markers

The development of protein omics technology has opened the door to the study of extracellular marker proteins in fungi. In mammals, transmembrane proteins, cell-specific proteins and cysteine protease inhibitors are usually chosen as biomarkers for corresponding research [31]; Protein markers in fungal EVs usually select protein which is enriched in EVs or exists only in EVs.Therefore, when looking for protein as a marker, it is necessary to compare and analyze fungal cells and protein in EVs to find a suitable protein [36] (Figure 1E).

#### 2.5.1. Sur 7 Family

Sur 7 is a four-span protein which is detected in many fungi such as *Candida albicans,* Saccharomyces cerevisiae, etc. It is an important part of MCC in the plasma membrane domain and plays an important role in the growth and development of fungi [88,89]. Sur 7 mainly affects the formation of cell walls and the growth and formation of hyphae, and some studies have shown that it also affects the invasion, toxicity and stress resistance of fungi [90,91]. It is an essential protein in fungi.

Sur 7 family proteins have been detected in the EVs of *Candida albicans* and *Cryptococcus*. It is thought that they can be used as biomarkers and effective tools to study the biogenesis and loading of EVs [35,37]. Markers are an important tool to study the function of fungal vesicles and play an important role in clarifying the mechanism of fungi. Sur 7 has also been found in *Colletotrichum higginsianum* [44], but it cannot be used as a reference for the time being, because due to the extraction scheme, the sample does not have enough peptide numbers or enough repetition. However, Sur 7 does not exist in every fungus. For example, Sur 7 has not been detected in the EVs of *Fusarium oxysporum* [36]. However, due to the fact that Sur 7 has been detected in many fungal EVs, it is believed that Sur 7 has great research value in fungal EV protein markers [37,49,73,92,93].

#### 2.5.2. Other Marker Proteins

In addition to the Sur 7 family, researchers have also found a suitable protein in the EVs of some plant pathogens by means of proteomics. Two kinds of EVs with different densities have been isolated by gradient purification with iodixanol, and membrane proteins of members of the SNARE family co-existing in the two kinds of vesicles have been selected. This protein family is highly conserved in structure and has similar functions in plasma membrane transport, so it has been selected as a marker of vesicle membrane by researchers. It has been found that this fluorescent signal is concentrated at the top of mycelia and could be used as a marker protein [44]. The protein of *Fusarium graminearum* EVs is similar to the protein predicted by *Candida albicans*. Surprisingly, through protein comparison analysis, CDC 42, RHO 3 and YKT 5 were found to be unique to EVs [36]. Due to their particularity, it is speculated that they could be used as a candidate gene for the EV protein marker of *Fusarium graminearum*. Many kinds of proteins that can be used as markers have also been found in *Fusarium oxysporum*, similar to *Fusarium graminearum* [36], but at present, due to the lack of data, it is impossible to support the study of common markers in Fusarium EVs. The study of markers can promote the study of fungi in biological interaction better. At present, there are still many mysteries about the mechanism of fungi, and the development of markers can make the mechanism of EVs more intuitive. To sum up, markers are of great significance in explaining the functions of fungi and are an important tool to explore biological interaction.

### 2.6. Role of Protein in Vaccine Research and Development

**Figure 3:**
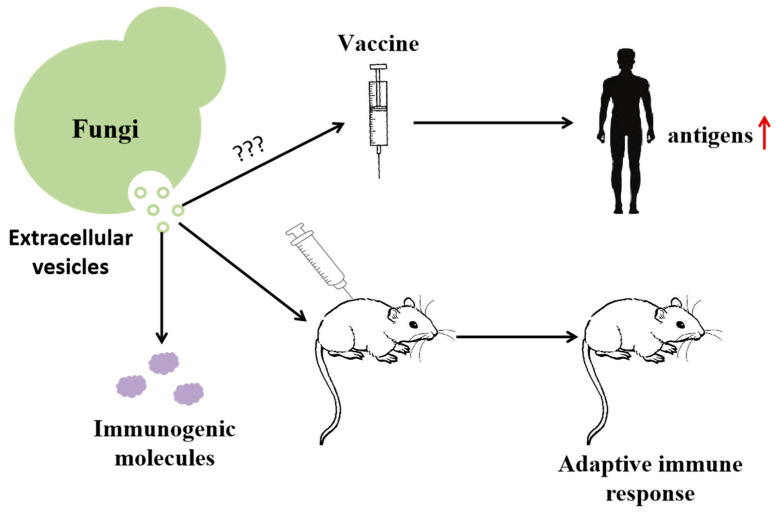
Role of protein in vaccine research and development.

A vaccine is a weakened or inactivated part (antigen) of a specific organism, which can trigger an immune response in vivo. In the field of vaccine research and development, in addition to the traditional inactivated vaccine, attenuated vaccines and other vaccine types, nanoparticle vaccines, DNA/RNA vaccines and other emerging vaccines are also being further studied [94]. In the process of vaccine development, it is necessary to find antigen factors which can effectively trigger protective immunity and improve lasting immunity. EVs interact with the host in the process of fungal infection, making the host produce an adaptive immune response, which meets the requirements of vaccine preparation and has high application prospects in the future [5]. At present, immunogenic proteins have been found in the EVs of human pathogenic bacteria such as *Candida albicans*, *Cryptococcus capsulatus*, *Paracoccus brazil*, etc. However, only Candida albicans and Cryptococcus have been reported in the research on EVs as vaccines (Figure 1D and Figure 3). Therefore, this paragraph is mainly aimed at the role of vesicular proteins of *Candida albicans* and *Cryptococcus* in immunity.

The EVs of fungal pathogens can cause immune response in mice, which may be related to immunogenic factors contained in the vesicles. This characteristic can be used in vaccine development. At present, it is not clear how to make EVs into vaccine and what kind of vaccine to make, so it is used ‘???’ replace. The increase of antigen in vivo after vaccination can improve immunity.

#### 2.6.1. *Candida albicans*

*Candidiasis* is a common fungal infection, with hundreds of thousands of cases reported each year. Candidemia, as the most common manifestation, has a high mortality rate due to the lack of effective treatment methods [95,96]. In the digestive system, *Candida albicans* acts as a commensal fungus and opportunistic pathogen, maintaining the stability of the intestinal microbiota through mutual inhibition and interaction with bacteria. However, excessive use of antibiotics disrupts the balance of the intestinal microbiota, leading to an overgrowth of *Candida albicans*. The fungus can then enter the bloodstream from the intestine, resulting in more severe disease conditions [97,98,99,100]. This type of infection is persistent and recurrent, causing significant impactd on human health, and its treatment is still under continuous research [101].

Immunogenic molecules have been found in the EVs of *Candida albicans*, suggesting that EVs have the potential to be used as immune modulators [102]. Based on this, EVs can serve as carriers of antigens associated with lipid bilayers or vesicle cores, such as the Bgl2 factor, which plays an important role in immune experiments [1,103]. Different species of *Candida albicans* EVs have different immunogenic proteins, including Adh1 in *Candida parapsilosis* and Eno1 and Tdh3 in *Candida tropicalis* [1]. These proteins are considered important targets for future vaccine research and may provide different preventive and therapeutic strategies for diseases caused by different Candida species.

#### 2.6.2. *Cryptococcus*

Cryptococcosis is a common fungal infection, with pulmonary cryptococcosis and central nervous system cryptococcosis being the most severe symptoms, mainly affecting immunocompromised individuals [104]. There are also rare cases of cryptococcosis, such as cutaneous cryptococcosis, vertebral cryptococcosis, renal cryptococcosis and laryngeal cryptococcosis [105,106,107,108,109]. However, due to limitations in tools, the diagnosis of cryptococcosis is not yet perfect, and our understanding of the disease is not thorough enough, which greatly hinders its treatment [110].

Vep is a vesicle-enriched protein, and its function is rarely reported on at present, but it has been selected as a candidate vaccine protein for cryptococcosis after testing [37,111]. Surprisingly, a study in 2021 discovered Vep in the EVs of *Cryptococcus neoformans* through proteomics, suggesting the potential use of EVs for vaccine preparation [37,72]. Juliana Rizzo found through cryo-electron microscopy that the surface structure of fungal EVs is similar to spike complexes on the viral envelope, suggesting their potential for vaccine research. Mouse immunization experiments have shown that EVs can induce adaptive immune responses even without adjuvants or carriers [68], indicating the great potential of vesicles in vaccine applications. In addition, *Cryptococcus neoformans* with Δcap59/Δsg11 mutations has been found to have characteristics suitable for attenuated vaccines [62], suggesting their great potential in vaccine development.

## 3. Summary and Prospect

EVs play an intermediary role in the transmembrane communication between fungi and other organisms. They transmit protein, RNA and other bioactive substances and play a very important role in the function of fungi. Proteins in EVs have a series of functions, such as transmitting information, constructing cell walls and participating in redox pathways. Fungi have different functions when interacting with other organisms. When fungi act, proteins in EVs can be used as a marker to show the mechanism more intuitively.

The proteins in some EVs are immunogenic and can be used to prepare vaccines. However, there are still many challenges to be faced in vaccine preparation. What we still need to overcome is the problem of EVs output. Among the present extraction methods, density gradient centrifugation, chromatography, solid medium centrifugation and the protoplast method are widely used. However, these schemes still cannot overcome the problem of low output and cannot achieve large-scale input and output for the time being. In addition, the first problem to be solved is to study the specific mechanism of the immune response triggered by EVs and to find out the key factors in this mechanism. Perhaps nano-carriers can be prepared to realize the mass production of fungal vaccines to reduce the occurrence of diseases. There are many kinds of human mycoses, and most of the research on human pathogenic fungi is still in its infancy and needs continuous research to provide theoretical and technical support for ensuring human safety.

Based on the multiple functions of protein in fungal EVs, we have reason to think that EVs play an important role in fungi, which is of great research value in studying the interaction mechanism between fungi and other organisms and is conducive to better utilization of fungi. However, more data and theoretical support are needed to discuss the mechanism of EVs. Because the diameter of EVs is between 40–150 nm and their volume is small, the yield of the EVs of fungi is low. In addition, due to the diversity of fungal secretions, the efficient purification of EVs is still a difficult problem to be solved as soon as possible. Of course, in view of the rich variety of protein species in casing, they can be widely used in agricultural plant disease prevention and treatment, medical disease treatment and other fields. Through the understanding and utilization of protein composition in EVs, EV preparation can be better applied to the prevention and treatment of diseases, which needs more experimental data to support and further explore through specific mechanisms. With the development of protein omics, we can understand the species of protein in fungi more intuitively, and lay a foundation for exploring the specific function of protein and the mechanism of action of fungi, which will gradually make it possible to prevent and treat diseases by using protein in fungi.
